# Chemoradiotherapy in geriatric patients with squamous cell carcinoma of the esophagus: Multi-center analysis on the value of standard treatment in the elderly

**DOI:** 10.3389/fonc.2023.1063670

**Published:** 2023-03-03

**Authors:** Tilman Bostel, Sati Akbaba, Daniel Wollschläger, Arnulf Mayer, Eirini Nikolaidou, Markus Murnik, Simon Kirste, Alexander Rühle, Anca-Ligia Grosu, Jürgen Debus, Christian Fottner, Markus Moehler, Peter Grimminger, Heinz Schmidberger, Nils Henrik Nicolay

**Affiliations:** ^1^ Department of Radiation Oncology, University Medical Center Mainz, Mainz, Germany; ^2^ German Cancer Consortium (Deutsches Konsortium fur Translationale Krebsforschung - DKTK) Partner Site Mainz, German Cancer Research Center (Deutsches Krebsforschungszentrum - DKFZ), Heidelberg, Germany; ^3^ Institute of Medical Biostatistics, Epidemiology and Informatics (IMBEI), University Medical Center Mainz, Mainz, Germany; ^4^ Department of Radiation Oncology, University of Freiburg – Medical Center, Freiburg, Germany; ^5^ German Cancer Consortium (Deutsches Konsortium fur Translationale Krebsforschung - DKTK) Partner Site Freiburg, German Cancer Research Center (Deutsches Krebsforschungszentrum - DKFZ), Heidelberg, Germany; ^6^ Department of Radiation Oncology, University Hospital of Heidelberg, Heidelberg, Germany; ^7^ German Cancer Consortium (Deutsches Konsortium fur Translationale Krebsforschung - DKTK) Partner Site Heidelberg, German Cancer Research Center (Deutsches Krebsforschungszentrum - DKFZ), Heidelberg, Germany; ^8^ Department of Internal Medicine I, University Medical Center Mainz, Mainz, Germany; ^9^ Department of General, Visceral and Transplant Surgery, University Medical Center Mainz, Mainz, Germany; ^10^ Department of Radiation Oncology, University of Leipzig Medical Center, Leipzig, Germany

**Keywords:** esophageal cancer, elderly patients, squamous cell cancer, chemoradiation, radiotherapy

## Abstract

**Background and purpose:**

To evaluate the tolerability and outcomes of chemoradiation in elderly patients with locally advanced esophageal squamous cell carcinoma (ESCC).

**Materials and methods:**

This multi-center retrospective analysis included 161 patients with SCC of the esophagus with a median age of 73 years (range 65-89 years) treated with definitive or neoadjuvant (chemo)radiotherapy between 2010 and 2019 at 3 large comprehensive cancer centers in Germany. Locoregional control (LRC), progression-free survival (PFS), distant metastasis-free survival (DMFS), overall survival (OS), and treatment-associated toxicities were analyzed, and parameters determining patient outcomes and treatment tolerance were assessed.

**Results:**

The delivery of radiotherapy without dose reduction was possible in 149 patients (93%). In 134 patients (83%), concomitant chemotherapy was initially prescribed; however, during the course of therapy, 41% of these patients (n = 55) required chemotherapy de-escalation due to treatment-related toxicities. Fifty-two patients (32%) experienced higher-grade acute toxicities, and 22 patients (14%) higher-grade late toxicities. The 2-year LRC, DMFS, PFS, and OS rates amounted to 67.5%, 33.8%, 31.4%, and 40.4%, respectively. Upon multivariate analysis, full-dose concomitant chemotherapy (vs. no or modified chemotherapy) was associated with significantly better DMFS (p=0.005), PFS (p=0.005) and OS (p=0.001). Furthermore, neoadjuvant chemoradiotherapy followed by tumor resection (vs. definitive chemoradiotherapy or definitive radiotherapy alone) significantly improved PFS (p=0.043) and OS (p=0.049). We could not identify any clinico-pathological factor that was significantly associated with LRC. Furthermore, definitive (chemo)radiotherapy, brachytherapy boost and stent implantation were significantly associated with higher-grade acute toxicities (p<0.001, p=0.002 and p=0.04, respectively). The incidence of higher-grade late toxicities was also significantly associated with the choice of therapy, with a higher risk for late toxicities when treatment was switched from neoadjuvant to definitive (chemo)radiotherapy compared to primary definitive (chemo)radiotherapy (p<0.001).

**Conclusions:**

Chemoradiation with full-dose and unmodified concurrent chemotherapy has a favorable prognostic impact in elderly ESCC patients; however, about half of the analyzed patients required omission or adjustment of chemotherapy due to comorbidities or toxicities. Therefore, the identification of potential predictive factors for safe administration of concurrent chemotherapy in elderly ESCC patients requires further exploration to optimize treatment in this vulnerable patient cohort.

## Introduction

Esophageal cancer is one of the most common cancers worldwide with over 470,000 new cases per year with a rapidly rising incidence (1, 2). Globally, most esophageal cancers are squamous cell carcinomas (ESCC) due to the widespread prevalence of risk factors. Despite all the advances in treatment in recent years, esophageal cancer remains one of the deadliest cancers globally due to an early lymphatic and vascular dissemination of tumor cells, with very poor 5-year survival rates ranging from 15-25% (2).

Increased age at diagnosis and an increasing life expectancy of the population in Western countries pose a problem for the treatment of esophageal cancer in the elderly, as treatment choices are governed by comorbidities, patient performance status and patient priorities (3, 4). In many landmark trials defining the role of chemoradiation in esophageal cancer, older patients have been underrepresented or excluded, making extrapolation of trial data to the elderly population problematic (5, 6). To date, there is no internationally consented definition of elderly patients; however, most studies define an age between 60 to 70 years as the minimum age for the classification of the elderly. For many elderly patients with esophageal cancers not suitable for surgical treatment, definitive radiotherapy with or without concomitant chemotherapy remains the curative treatment of choice. Despite the demonstrated benefits of adding chemotherapy to radiotherapy, concomitant chemoradiotherapy can result in severe adverse effects, especially in case of comorbidities or poor performance status prior to treatment initiation (7).

To date, there are only few datasets available that investigated the benefit of standard chemoradiotherapy for the treatment of esophageal cancers in the elderly (7–9). Our study aimed to analyze toxicity profiles and oncologic outcomes in a large multi-center cohort of elderly ESCC patients treated with neoadjuvant or definitive (chemo)radiotherapy. We also investigated potential prognostic factors associated with an adverse treatment response and the occurrence of higher-grade toxicities in order to guide treatment decisions in this vulnerable patient population.

## Material and methods

### Patients

In this retrospective multi-center study, patients with histologically confirmed ESCC and a minimum age of 65 years without distant metastasis at initial diagnosis were included. Patients were treated with either chemoradiotherapy or radiotherapy at the University Hospitals of Mainz, Freiburg, and Heidelberg from 2000 to 2019. Demographic, clinical and pathological data were obtained from electronic medical records, pathology reports and the cancer registries of participating centers. Staging of esophageal carcinomas was based on the versions of the TNM classification (Union for International Cancer Control [UICC]) and the clinical stages of the American Joint Committee on Cancer (AJCC) that were current at the time of first diagnosis (i.e., 6^th^, 7^th^ or 8^th^ edition of the UICC-AJCC TNM classification). This analysis has been approved by the independent ethics committees of the medical faculties of the universities of Mainz (no reference number), Freiburg (reference no. 275/18) and Heidelberg (reference no. S-040/2018).

### Treatment groups

The majority of patients were treated for locally advanced tumors and received either neoadjuvant chemoradiotherapy followed by surgery or definitive (chemo)radiotherapy. Treatment decisions were based on multidisciplinary tumor board recommendations. Radiation planning was performed with either conventional 3D conformal radiotherapy (3D-CRT) or intensity-modulated radiotherapy (IMRT).

A total of 61 patients received (chemo)radiation in neoadjuvant intention with a median total dose of 41.4 Gy (range 40.0 - 56.0 Gy) and median single doses of 1.8 Gy (range 1.8 - 2.0 Gy). Only four of the preoperatively treated patients received sequential dose escalation to the macroscopic tumor by either teletherapy (n = 3, cumulative doses of 50.4 - 54 Gy, single doses 1.8 - 2.1 Gy) or brachytherapy (n = 1, cumulative dose of 54 Gy, single dose 4.0 Gy). One patient underwent additional postoperative irradiation due to incomplete tumor resection (R1 situation) with a cumulative dose of 66 Gy. In 8 patients, neoadjuvant chemoradiotherapy was not followed by surgery because of treatment-related deterioration of patient performance status (n = 2), patient refusal to undergo surgery after completion of neoadjuvant therapy (n = 4), comorbidities (n = 1) or newly diagnosed liver metastases upon intermediate staging prior to surgery (n = 1). In addition, 11 patients switched from the planned neoadjuvant chemoradiation to a definitive treatment regimen by increasing the doses of both radiotherapy and chemotherapy because of patient refusal to undergo surgery (n = 1), irresectability (n = 6), comorbidities (n = 2) and for unknown reasons (n = 2). In our analysis, we assigned all those patients with initial neoadjuvant therapy and no subsequent surgery to the definitive (chemo)radiation group. Neoadjuvant chemoradiotherapy concepts applied until 2012 differed between participating centers (see **Supplementary Table S1**). From 2013 onwards, neoadjuvant treatments were performed according to the protocol of the CROSS trial at all participating centers. The CROSS regimen comprises radiotherapy up to a total dose of 41.4 Gy using single doses of 1.8 Gy and concurrent application of paclitaxel (50 mg/m^2^) and carboplatin (AUC of 2 mg/ml/min) on days 1, 8, 15, 22 and 29 (n = 19 patients) (6).

Definitive (chemo)radiotherapy was administered to 119 patients. Primary tumors and affected lymph nodes including a safety margin (*see below*) and the regional lymphatic drainage area (elective) were treated to a median total dose of 50 Gy (range 12.6 - 73.8 Gy) using median single doses of 1.8 Gy (range 1.6 - 2.5 Gy). The majority of patients (n = 86, 72%) received dose escalation to the macroscopic tumor tissue by using simultaneous integrated or sequential teletherapy boost (median total dose 9.0, range 4.0 - 27.0 Gy; median single dose 2.0 Gy, range 1.8 - 3.0 Gy; n = 78, 66%) and/or brachytherapy boost (median total dose 8.0, range 4.0 - 24.0 Gy; median single dose 4.0 Gy, range 4.0 - 6.0 Gy; n = 24, 20%). The median cumulative dose was 58.8 Gy (range 12.6 - 74.0 Gy). Different chemotherapy regimens were applied in combination with definitive radiotherapy, as outlined in **Supplementary Table S2**.

The fitness of patients to receive radiotherapy and concomitant chemotherapy was assessed at baseline. Reasons for discontinuation or reduction of radiotherapy and concomitant chemotherapy were obtained from patient files. For this analysis, we defined combinations of a platinum derivate and 5-fluorouracil (5-FU), carboplatin and paclitaxel, mitomycin C and 5-FU and FOLFOX as standard chemotherapy regimens. Other chemotherapy regimens such as monotherapy with 5-FU, Capecitabine or a platinum compound alone or dose reduction of chemotherapy during radiation treatment were defined as a modification of chemotherapy. Moreover, we defined full-dose radiotherapy and full-dose chemotherapy as administration of both treatment modalities without interruption, dose reduction or modification.

### Target volume definition

The primary gross tumor volume (GTV) and lymph node GTV(s) were defined based on planning computed tomography (CT) and staging examinations including contrast-enhanced CT, PET/CT, endosonography, and endoscopic clip markings of the oral and aboral tumor margins, if available. The clinical target volume (CTV) was generated by adding a safety margin of 3 - 5 cm in the oral and aboral directions and 1 - 2 cm in the axial direction to the GTV of the primary tumor and 1 cm safety margin to the GTV of the lymph node metastasis. Regional elective lymphatic drainage was regularly included in the CTV. Adjustment of the CTV to anatomical barriers such as the bone, lungs, or heart and to the stomach was performed in case of distal cancers. The planning target volume (PTV) included the CTV and an additional craniocaudal and lateral safety margin of 0.5 - 1.0 cm. Boost volumes were obtained by expanding the primary GTV by 2 cm craniocaudally and 1 - 2 cm circumferentially, and the lymph node GTV(s) by 0.5 - 1 cm in all directions.

### Oncologic outcomes and toxicity

All patients received regular follow-up examinations at 3- to 6-month intervals, including clinical examinations as well as multi-region CT imaging. In case of suspected locoregional or distant tumor recurrence on CT, additional diagnostic work-up was performed. Locoregional control (LRC) was defined as the time from the end of radiotherapy without progression of the primary tumor and without evidence of new-onset or progressive locoregional lymph node metastases. Distant metastasis-free survival (DMFS) was defined as the time from the end of radiotherapy to the new onset of distant metastases or death from any cause. Overall survival (OS) was defined as the time from the end of radiotherapy to death from any cause. Progression-free survival (PFS) was defined as the time from the end of radiotherapy to progression of tumor disease of any site or death from any cause. Missing survival data were obtained from the cancer registries. Acute and chronic adverse events were classified according to the CTCAE criteria version 5.0.

### Statistical analysis

Statistical analysis was performed using R software, version 4.1.3 (R Core Team 2022, Vienna, Austria). P-values of p<0.05 were considered statistically significant. The Kaplan-Meier method was used to estimate survival after radiotherapy, with the log-rank test to determine statistical significance. Moreover, multivariable analyses were performed using the Cox proportional hazards model and associated Wald tests to identify predictors of LRC, DMFS, PFS, and OS after radiotherapy. Since chemotherapy was sometimes completed after the end of radiotherapy, tests involving completion of chemotherapy as a predictor were based on a Cox model with time-varying covariates to avoid immortal-time bias.

## Results

### Patient and treatment characteristics

A total of 161 patients with histologically confirmed SCC of the esophagus were included in this retrospective multi-center analysis. Patients were predominantly male (n = 119, 74%) and had a median age of 73 years (range 65 to 89 years). According to the consensus definition of the United States National Institute of Aging, the study population was subdivided into the following 3 age groups: “young olds” (65 to 74 years), “older olds” (75 to 84 years) and “oldest olds” (≥ 85 years) (10). In our study population, the majority of patients belonged to the “young old” subgroup (n = 96, 60%), whereas the proportion of patients classified as “older old” and “oldest old” amounted to 37% (n = 59) and 4% (n = 6), respectively. The majority of analyzed patients exhibited a relatively good performance status prior to treatment, with 133 patients (83%) having Eastern Cooperative Oncology Group (ECOG) values in the range of 0 to 1. Most tumors were located in the thoracic portion of the esophagus (n = 142, 88%), and 37% (n = 59) of cancers were localized in the mid-thoracic segment (24 to 32 cm from dentition) with a median tumor length of 5 cm (range 1 - 13 cm). The majority of patients suffered from locally advanced disease at diagnosis with 134 patients (83%) having cT3/4 tumors and 119 patients (74%) exhibiting lymphogenic tumor spread on imaging or endosonography. The majority of SCC were moderately or poorly differentiated (55% and 31%, respectively).

Forty-two patients (26%) received neoadjuvant radiotherapy of whom 19 (45%) were treated with carboplatin and paclitaxel, and 23 patients (55%) with cisplatin and 5-FU. No patient had to prematurely discontinue neoadjuvant radiotherapy. Concomitant chemotherapy was reduced or modified in 12 of these patients (29%) due to deteriorating performance status or acute toxicities. Overall, more than 80% of the initially prescribed chemotherapy dose could be applied in 35 of the patients receiving neoadjuvant treatment (83%).

One hundred and nineteen patients (74%) were treated with definitive radiotherapy, of whom 92 patients (77%) received concurrent chemoradiotherapy and 4 patients (3%) received a concomitant EGFR receptor antibody (cetuximab). Various concurrent chemotherapy regimens were administered in the definitive treatment situation, including cisplatin/5-FU (n = 59, 50%), carboplatin/5-FU (n = 4, 3%), carboplatin/paclitaxel (n = 10, 8%), FOLFOX (n = 9, 8%), mitomycin C and 5-FU (n = 2, 2%), 5-FU or Capecitabine alone (n = 6, 5%), or a platinum derivative alone (n = 2, 2%). One hundred and seven patients (90%) received full dose definitive radiotherapy, and only 60 patients (50%) completed concomitant chemotherapy as initially prescribed. The reasons for premature discontinuation of radiotherapy were acute toxicities and deterioration of general condition (n = 8; 7%), patient request (n = 1, 1%), or death during treatment (n = 3; 3%), while chemotherapy dose was reduced due to treatment-related toxicities. The full treatment regimen of definitive chemoradiation including all concomitant and adjuvant chemotherapy cycles could only be administered to 49 patients (41%) due to treatment-related toxicities. In 74 patients (62%), more than 80% of initially prescribed chemotherapy dose was applied in the definitive treatment situation.

Overall, 48 patients (30%) underwent bougienage due to malignant stenosis of the esophagus and 29 patients (18%) underwent stent implantation.

Detailed information on tumor and patient characteristics are listed in **Table 1**.

**Table 1:** Tumor and patient characteristics at baseline.

Variable	Value	n	%
Gender	male	119	73.9
	female	42	26.1
Age	65-74 years	96	59.6
	75-84 years	59	36.7
	≥ 85 years	6	3.7
ECOG	0	58	36.0
	1	75	46.6
	2	28	17.4
Localization (distance from incisors)	Cervical (15 - 18 cm)	19	11.8
	Upper thoracic (18 - 24 cm)	42	26.1
	Middle thoracic (24 - 32 cm)	59	36.7
	Lower thoracic (32 - approximate 40 cm)	41	25.5
cT-stage	T1	6	3.7
	T2	19	11.8
	T3	101	62.7
	T4	33	20.5
	Tx	2	1.2
cN-stage	N0	40	24.8
	N+	119	73.9
	Nx	2	1.2
M-stage	M0	161	100.0
	M1	0	0
AJCC-stage	1	4	2.5
	2	39	24.2
	3	81	50.3
	4a	34	21.1
	NA	3	1.9
Grading	G1	3	1.9
	G2	92	57.1
	G3	48	29.8
	G4	0	0
	Gx	18	11.2
Charlson Comorbidity Index	≤ 5	64	39.8
	> 5	95	59.0
	NA	2	1.2

Staging of esophageal carcinomas was based on the versions of the TNM classification (Union for International Cancer Control [UICC]) and the clinical stages of the American Joint Committee on Cancer (AJCC) that were current at the time of first diagnosis (i.e., 6^th^, 7^th^ or 8^th^ edition of the UICC-AJCC TNM classification).

ECOG, Eastern Cooperative Oncology Group; AJCC, American Joint Committee on Cancer.

### Treatment outcome

Three patients died (2%) during radiotherapy due to sepsis (n = 2) or acute tumor bleeding (n = 1), respectively, and were therefore excluded from all further analyses regarding oncologic response. For the entire cohort, the 1-, 2- and 5-year LRC rates were 79.7% (95% CI 72.6% - 87.4%), 67.5% (95% CI 58.4% - 77.9%) and 54.7% (95% CI 44.2% - 67.7%), while the corresponding DMFS rates were 49.7% (95% CI 42.4% - 58.2%), 33.8% (95% CI 26.9% - 42.5%) and 15.9% (95% CI 10.2% - 24.8%), respectively. PFS after 1, 2 and 5 years amounted to 46.9% (95% CI 39.6% - 55.6%), 31.4% (95% CI 24.6% - 40.1%) and 15.5% (95% CI 9.7% - 24.6%), and OS to 58.2% (95% CI 50.9%-66.4%), 40.4% (95% CI 33.2%-49.1%) and 17.3% (95% CI 11.4%-26.3%) at the respective time points. The recurrence patterns are summarized in detail in **Supplementary Table S3**.

**Figure 1:**
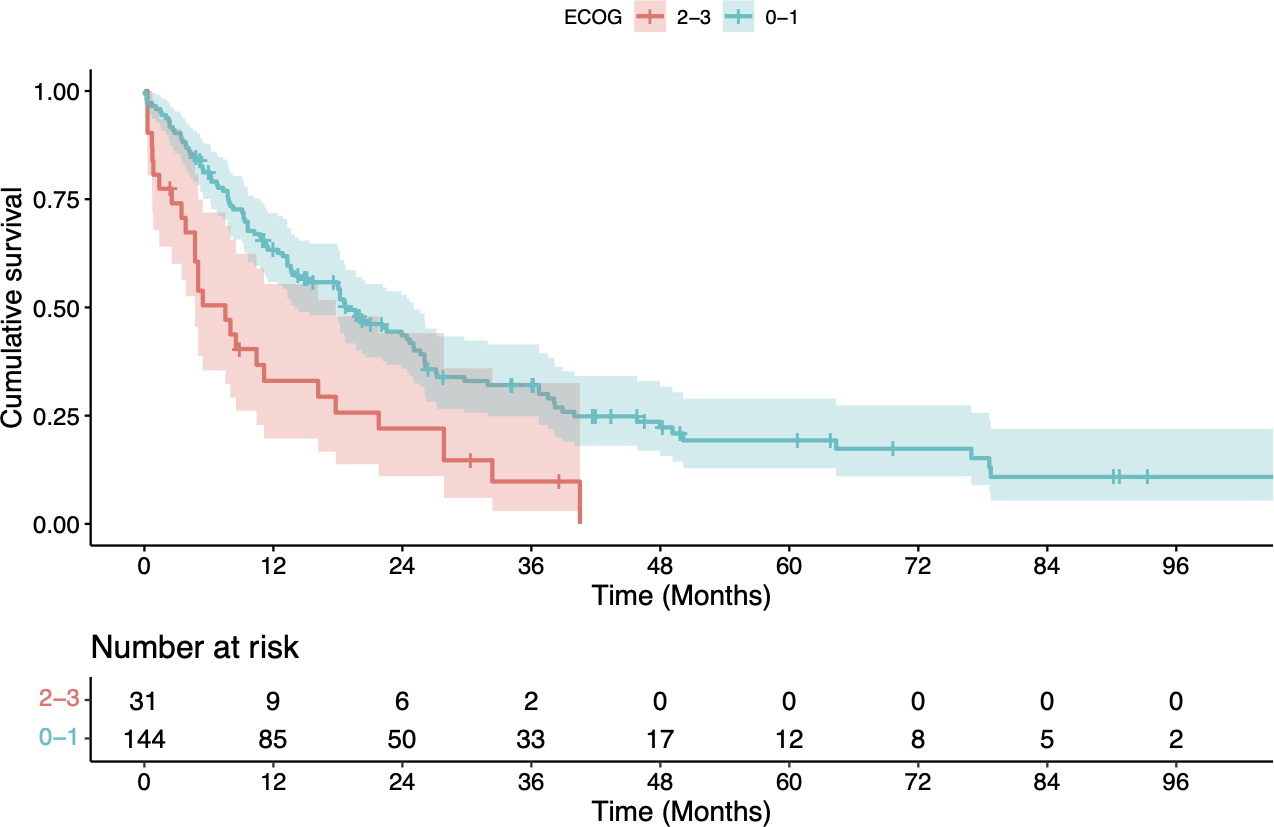
Kaplan-Meier estimate of overall survival (OS) after radiotherapy stratified by Eastern Cooperative Oncology Group (ECOG) Performance Score 0 - 1 vs. 2 - 3. OS was significantly better for patients with an ECOG of ≤ 1 (p = 0.001, log-rank test).

**Figure 2:**
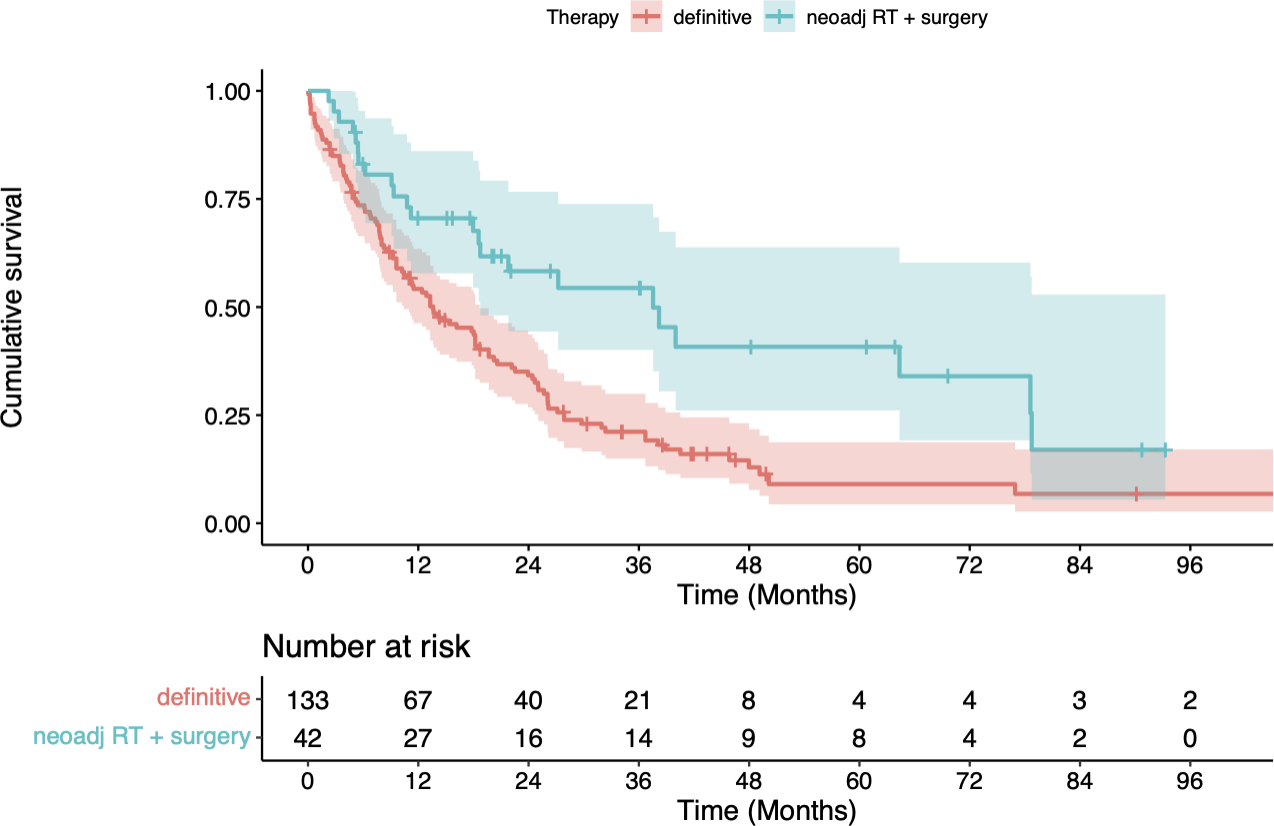
Kaplan-Meier estimate of overall survival (OS) after radiotherapy stratified by scheduled application of chemotherapy vs. no or modified administration of chemotherapy. OS was significantly better for patients with a scheduled application of chemotherapy (p < 0.001, log-rank test).

**Figure 3:**
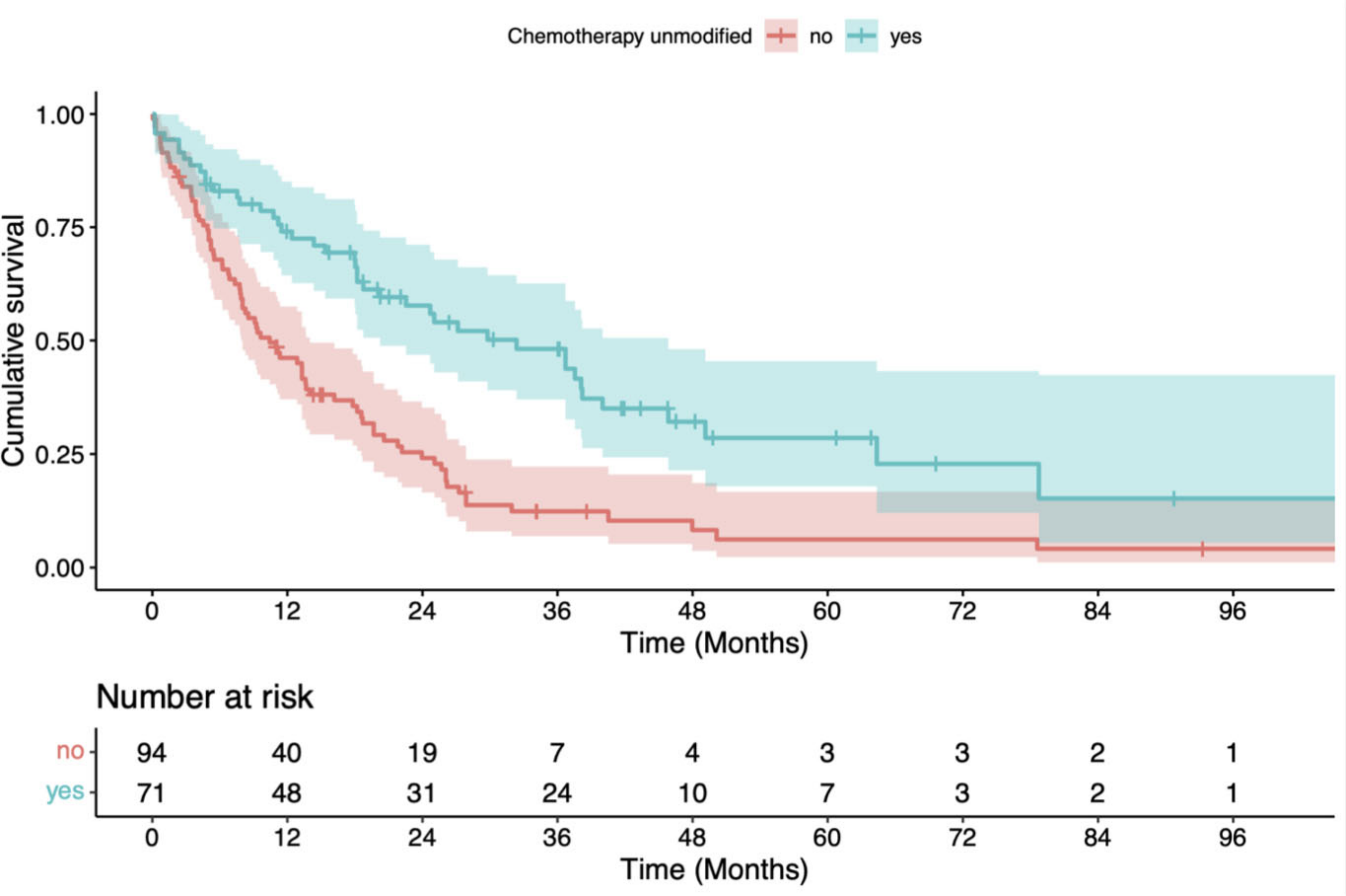
Kaplan-Meier estimate of overall survival (OS) after radiotherapy stratified by neoadjuvant chemoradiation followed by surgical resection vs. definitive treatment with (chemo)radiation. OS was significantly better for patients who received neoadjuvant treatment (p = 0.002, log-rank test).

Better OS was significantly associated with patient performance status (ECOG 1-2 vs. 3-4: p=0.001, log-rank test), administration of full-dose systemic therapy (vs. no or reduced systemic therapy doses; p<0.001, univariate Cox model) and neoadjuvant chemoradiation followed by surgery (vs. other treatments; p=0.002, log-rank test) (see **Table 2A** and **Figures 1**–**3**). For radiotherapy adherence (complete vs. incomplete administration), there was also a statistically significant OS difference after radiotherapy (p=0.01, log-rank test; 2-year OS 42.0% vs. 18.8%). Age, gender, comorbidities (Charlson Comorbidity Index), tumor extension (T stage), metastatic nodal spread (N stage), tumor stage according to the Union for International Cancer Control (UICC), and localization of the primary tumor were not significantly associated with OS (see **Table 2A**).

**Table 2A:** Univariate analyses of potential prognostic factors for overall survival (OS).

Factors	OS at 1 year (%)	OS at 2 years (%)	OS at 5 years (%)	p - value
Age				
65 - 74 years	61	47	25	
75 – 84 years	56	33	4	
≥ 85 years	40	–	–	0.08
Gender				
Female	61	32	13	
Male	57	43	19	0.60
ECOG score				
0 - 1	63	45	20	
2 - 3	33	21	–	**0.001**
Charlson Comorbidity Index				
≤ 5	59	40	24	
> 5	57	41	11	0.20
Clinical tumor classification (cT)				
1	67	50	–	
2	68	50	9	
3	56	40	20	
4	58	37	22	0.90
Clinical lymph node classification (cN)				
Nodal negative (N0)	48	35	11	
Nodal positive (N+)	62	43	21	0.10
Tumor stage (AJCC)				
1 - 2	53	36	9	
3	59	45	20	
4a	63	41	26	0.70
Localization of the primary tumor				
0 - 18 cm distance from the incisors (cervical)	68	51	24	
18 - 24 cm distance from the incisors (upper thoracic third)	66	49	25	
24 - 32 cm distance from the incisors (middle thoracic third)	58	32	11	
32 - 40 cm distance from the incisors (lower thoracic third)	46	38	22	0.60
Administration of full-dose RT				
yes	60	42	18	
no	28	19	–	**0.01**
Cumulative dose of RT (EQD2)				
≤ 50 Gy	57	42	21	
> 50 Gy	59	39	14	0.90
Administration of non-modified, full-dose chemotherapy				
yes	76	58	30	
no	43	24	7	**< 0.001**
Treatment concept				
Neoadjuvant CRT followed by surgical resection	71	58	41	
Definitive RT/CRT	54	34	9	**0.002**

ECOG, Eastern Cooperative Oncology Group; AJCC, American Joint Committee on Cancer; RT, radiotherapy; EQD2, equivalent dose in 2 Gy fractions; CRT, chemoradiotherapy. Bold values, significant p-values.

In multivariate analysis, neoadjuvant chemoradiotherapy followed by tumor resection and administration of concomitant non-modified chemotherapy remained significantly associated with better PFS and OS (2-year PFS 21.2% vs. 9.5%, HR 0.55, 95% CI 0.36 - 0.83, p=0.005; 2-year OS 48% vs. 15%; HR 0.51, 95% CI 0.34 - 0.77, p=0.001), while DMFS was only significantly associated with fully administered unmodified chemotherapy (see **Tables 2A**–**C** and **Tables 3A**–**C**). In contrast, none of the factors analyzed was statistically significantly associated with LRC (see **Tables 2D**, **3D**).

**Table 2B:** Univariate analyses of potential prognostic factors for progression-free survival (PFS).

Factors	PFS at 1 year (%)	PFS at 2 years (%)	PFS at 5 years (%)	p - value
Age				
65 – 74 years	57	37	20	
75 – 84 years	54	32	9	
≥ 85 years	21	–	–	0.08
Gender				
Female	54	26	12	
Male	55	37	16	0.60
ECOG score				
0 - 1	58	35	17	
2 - 3	37	26	6	0.06
Charlson Comorbidity Index				
≤ 5	59	39	25	
> 5	50	29	6	0.05
Clinical tumor classification (cT)				
1	67	67	25	
2	53	32	8	
3	53	34	16	
4	59	32	22	1.00
Clinical lymph node classification (cN)				
Nodal negative (N0)	50	31	14	
Nodal positive (N+)	56	35	16	0.50
Tumor stage (AJCC)				
1 - 2	46	27	12	
3	56	38	14	
4a	61	34	26	0.50
Localization of the primary tumor				
0 - 18 cm distance from the incisors (cervical)	52	34	15	
18 - 24 cm distance from the incisors (upper thoracic third)	67	44	23	
24 - 32 cm distance from the incisors (middle thoracic third)	54	30	9	
32 - 40 cm distance from the incisors (lower thoracic third)	44	29	19	0.80
Administration of full-dose RT				
yes	55	35	17	
no	45	11	–	**0.04**
Cumulative dose of RT (EQD2)				
≤ 50 Gy	55	35	18	
> 50 Gy	55	33	14	0.90
Administration of non-modified, full-dose chemotherapy				
yes	69	51	26	
no	40	19	8	0.47
Treatment concept				
Neoadjuvant CRT followed by surgical resection	71	55	36	
Definitive RT/CRT	49	27	8	**< 0.001**

ECOG, Eastern Cooperative Oncology Group; AJCC, American Joint Committee on Cancer; RT, radiotherapy; EQD2, equivalent dose in 2 Gy fractions; CRT, chemoradiotherapy. Bold values, significant p-values.

**Table 2C:** Univariate analyses of potential prognostic factors for distant metastasis-free survival (DMFS).

Factors	DMFS at 1 year (%)	DMFS at 2 years (%)	DMFS at 5 years (%)	p - value
Age				
65 – 74 years	50	39	22	
75 – 84 years	51	28	6	
≥ 85 years	25	–	–	0.30
Gender				
Female	50	24	12	
Male	50	37	17	0.50
ECOG score				
0 - 1	54	36	18	
2 - 3	30	21	–	**0.003**
Charlson Comorbidity Index				
≤ 5	50	38	23	
> 5	48	31	9	0.20
Clinical tumor classification (cT)				
1	67	50	–	
2	47	34	9	
3	47	34	17	
4	56	34	23	1.00
Clinical lymph node classification (cN)				
Nodal negative (N0)	39	30	10	
Nodal positive (N+)	54	36	19	0.1
Tumor stage (AJCC)				
1 - 2	38	25	8	
3	52	40	17	
4a	57	35	27	0.30
Localization of the primary tumor				
0 - 18 cm distance from the incisors (cervical)	53	41	15	
18 - 24 cm distance from the incisors (upper thoracic third)	63	40	27	
24 - 32 cm distance from the incisors (middle thoracic third)	49	30	9	
32 - 40 cm distance from the incisors (lower thoracic third)	36	30	20	0.80
Administration of full-dose RT				
yes	53	36	17	
no	9	–	–	**< 0.001**
Cumulative dose of RT (EQD2)				
≤ 50 Gy	46	34	19	
> 50 Gy	53	34	14	0.90
Administration of non-modified, full-dose chemotherapy				
yes	67	51	27	
no	34	18	7	**< 0.001**
Treatment concept				
Neoadjuvant CRT followed by surgical resection	61	55	36	
Definitive RT/CRT	46	27	9	**0.002**

ECOG, Eastern Cooperative Oncology Group; AJCC, American Joint Committee on Cancer; RT, radiotherapy; EQD2, equivalent dose in 2 Gy fractions; CRT, chemoradiotherapy. Bold values, significant p-values.

**Table 2D:** Univariate analyses of potential prognostic factors for locoregional control (LRC).

Factors	LRC at 1 year (%)	LRC at 2 years (%)	LRC at 5 years (%)	p - value
Age				
65 – 74 years	84	71	63	
75 – 84 years	76	64	40	
≥ 85 years	–	–	–	**0.03**
Gender				
Female	84	60	43	
Male	78	68	57	0.90
ECOG score				
0 - 1	82	70	58	
2 - 3	64	51	–	0.05
Charlson Comorbidity Index				
≤ 5	86	71	64	
> 5	75	65	46	0.20
Clinical tumor classification (cT)				
1	50	50	–	
2	79	79	68	
3	80	66	51	
4	86	66	66	0.20
Clinical lymph node classification (cN)				
Nodal negative (N0)	63	50	35	
Nodal positive (N+)	85	73	61	**0.009**
Tumor stage (AJCC)				
1 - 2	64	58	45	
3	87	71	54	
4a	82	71	71	0.20
Localization of the primary tumor				
0 - 18 cm distance from the incisors (cervical)	92	81	58	
18 - 24 cm distance from the incisors (upper thoracic third)	79	63	47	
24 - 32 cm distance from the incisors (middle thoracic third)	84	71	58	
32 - 40 cm distance from the incisors (lower thoracic third)	69	62	62	0.60
Administration of full-dose RT				
yes	80	69	56	
no	75	–	–	0.50
Cumulative dose of RT (EQD2)				
≤ 50 Gy	81	69	69	
> 50 Gy	79	67	46	0.20
Administration of non-modified, full-dose chemotherapy				
yes	88	79	67	
no	73	55	45	**0.02**
Treatment concept				
Neoadjuvant CRT followed by surgical resection	91	83	77	
Definitive RT/CRT	75	62	46	**0.009**

ECOG, Eastern Cooperative Oncology Group; AJCC, American Joint Committee on Cancer; RT, radiotherapy; EQD2, equivalent dose in 2 Gy fractions; CRT, chemoradiotherapy. Bold values, significant p-values.

**Table 3A:** Multivariate analysis of clinical parameters regarding overall survival (OS) of esophageal SCC in elderly patients after definitive or neoadjuvant CRT/RT.

Factors	HR	CI 95%	p-value
Age	1.003	0.965 - 1.042	0.892
ECOG score	1.537	0.946 - 2.497	0.083
Administration of full-dose RT	0.752	0.380 - 1.488	0.414
Administration of non-modified, full-dose chemotherapy	0.509	0.336 - 0.771	**0.001**
Neoadjuvant CRT followed by surgery vs. definitive RT/CRT	0.608	0.370 - 0.999	**0.049**

SCC, squamous cell carcinoma; CRT, chemoradiotherapy; RT, radiotherapy; ECOG, Eastern Cooperative Oncology Group. Bold values, significant p-values.

**Table 3B:** Multivariate analysis of clinical parameters regarding progression-free survival (PFS) of esophageal SCC in elderly patients after definitive or neoadjuvant CRT/RT.

Factors	HR	CI 95%	p-value
Age	0.998	0.960 - 1.037	0.913
ECOG score	1.475	0.897 - 2.425	0.125
Administration of full-dose RT	0.832	0.386 - 1.793	0.639
Administration of non-modified, full-dose chemotherapy	0.548	0.361 - 0.831	**0.005**
Neoadjuvant CRT followed by surgery vs. definitive RT/CRT	0.597	0.362 - 0.983	**0.043**

SCC, squamous cell carcinoma; CRT, chemoradiotherapy; RT, radiotherapy; ECOG, Eastern Cooperative Oncology Group. Bold values, significant p-values.

**Table 3C:** Multivariate analysis of clinical parameters regarding distant metastasis-free survival (DMFS) of esophageal SCC in elderly patients after definitive or neoadjuvant CRT/RT.

Factors	HR	CI 95%	p-value
Age	0.994	0.957 - 1.032	0.742
ECOG score	1.364	0.831 - 2.240	0.219
Administration of full-dose RT	0.596	0.303 - 1.172	0.134
Administration of non-modified, full-dose chemotherapy	0.544	0.357 - 0.829	**0.005**
Neoadjuvant CRT followed by surgery vs. definitive RT/CRT	0.621	0.376 - 1.025	0.063

SCC, squamous cell carcinoma; CRT, chemoradiotherapy; RT, radiotherapy; ECOG, Eastern Cooperative Oncology Group; CCI, Charlson Comorbidity Index. Bold value, significant p-value.

**Table 3D:** Multivariate analysis of clinical parameters regarding locoregional control (LRC) of esophageal SCC in elderly patients after definitive or neoadjuvant CRT/RT.

Factors	HR	CI 95%	p-value
Age	1.053	0.987 - 1.120	0.120
ECOG score	1.736	0.739 - 4.080	0.210
Administration of full-dose RT	1.014	0.226 - 4.540	0.990
Administration of non-modified, full-dose chemotherapy	0.569	0.279 - 1.160	0.120

SCC, squamous cell carcinoma; CRT, chemoradiotherapy; RT, radiotherapy; ECOG, Eastern Cooperative Oncology Group.

### Treatment-related toxicities

In our study population, 51 patients (32%) developed severe or life-threatening acute toxicities (CTCAE grade 3/4) during (chemo)radiation, with hematologic side effects, new-onset or progressive dysphagia with consecutive weight loss and increasing esophageal stenosis being the most common adverse events. Acute grade 5 toxicities with lethal outcome were observed in 3 patients (2%).

Higher-grade late toxicities (CTCAE grade 3/4) were diagnosed in 22 patients (14%), with dysphagia and/or new-onset or increasing stenosis of the esophagus as the most prevalent adverse events that required further interventions. Two patients (1%) developed late grade 5 toxicities with ulceration in the anastomotic area, recurrent bleeding and fatal outcome. The detailed toxicity profile of radiation or chemoradiation treatment is summarized in **Table 4**. In our analysis, acute toxicity was significantly associated with the type of therapy, with neoadjuvant chemoradiotherapy associated with a lower chance for the occurrence of severe acute toxicity than definitive (chemo)radiotherapy (p<0.001; see **Supplementary Table S4**). In addition, brachytherapy boost and stent implantation were associated with a higher risk for severe acute toxicities. Other factors such as age and sex, performance status, comorbidities, location or length extent of the primary tumor, T or N stage, UICC stage, or administration of concurrent chemotherapy without dose reduction were not statistically significantly associated with the occurrence of severe acute toxicities (see **Supplementary Table S4**).

**Table 4:** Acute and late severe and life-threatening toxicities (grade 3 and 4 according to CTCAE v5.0) of (chemo)radiotherapy in elderly patients with SCC of the esophagus.

Variable	Value
**Acute toxicities – no. (%)**	51 (31.7)
- Hematological side effects – no. (%)	27 (16.8)
- Dysphagia – no. (%)	27 (16.8)
- Esophageal stenosis – no. (%)	9 (5.6)
- Mucositis/odynophagia – no. (%)	10 (6.2)
- Acute renal failure – no. (%)	2 (1.2)
- Tumor bleeding – no. (%)	4 (2.5)
- Fistula – no. (%)	1 (0.6)
- Pneumonia – no. (%)	2 (1.2)
- Perforation and mediastinitis – no. (%)	1 (0.6)
- Pulmonary artery embolism – no. (%)	2 (1.2)
- Damage to the vestibular organ – no. (%)	1 (0.6)
- Radiation dermatitis – no. (%)	1 (0.6)
**Late toxicities – no. (%)**	22 (13.7)
- Dysphagia – no. (%)	19 (11.8)
- Esophageal stenosis – no. (%)	18 (11.2)
- Ulcera with tumor bleeding and lethal outcome – no. (%)	2 (1.2)
- Diarrhea – no. (%)	1 (0.6)

The incidence of severe late toxicities was also significantly associated with the type of therapy, with a higher chance of late toxicities when there was a switch in the treatment concept from neoadjuvant to definitive (chemo)radiotherapy compared with primary definitive (chemo)radiotherapy (p< 0.001), while no statistically significant association was found for the age, sex, comorbidities, localization of the primary tumor, N stage, stent implantation, brachytherapy, and chemotherapy without dose reduction (see **Supplementary Table 5**).

## Discussion

Our analysis demonstrated very good tolerability of radiotherapy in elderly esophageal squamous cell cancer patients. However, only about half of patients in our cohort could receive concomitant chemotherapy without dose reduction or modification due to comorbidities and toxicities. Upon multivariate analysis, neoadjuvant chemoradiotherapy followed by tumor resection and concomitant non-modified chemotherapy was found to be the key factor determining better PFS and OS in elderly ESCC patients.

In general, therapeutic decisions in the treatment of elderly cancer patients depend to a considerable extent on patient-individual factors such as patient performance, comorbidities, and chronological age of patients. Many studies have shown that age-related modifications of standard therapy in general influence treatment response of various cancers (11, 12).

However, despite the high clinical importance, available data for esophageal cancer therapy in the elderly are mainly derived from retrospective studies except for one recent prospective randomized study that reported survival of elderly esophageal cancer patients after chemoradiotherapy with S-1 or radiotherapy alone (8, 9, 13–17). Most reports analyzed elderly esophageal cancer patients with both adenocarcinomas and SCCs, and most of these studies have demonstrated a survival benefit for additional concomitant chemotherapy, including the only published prospective study to date with an exclusively elderly patient population (9). For example, in a large retrospective analysis of the SEER database, 3020 elderly patients (≥ 65 years) with esophageal cancers treated with chemoradiation or radiotherapy alone were analyzed. In this analysis, the five-year overall and cancer-specific survival rates were only 13% and 20%, respectively; comparing the treatment modalities by propensity-score matching, a significant survival benefit of chemoradiotherapy versus radiotherapy was evident regardless of patient age (8). Other retrospective studies of elderly patients with esophageal cancer reported 5-year OS rates in the range of 5 - 36% after (chemo)radiotherapy (17, 18).

In this large multi-center cohort focusing on ESCC, we demonstrated that radiotherapy and chemoradiation are feasible treatment modalities for elderly esophageal cancer patients associated with relatively high rates of LRC (2- and 5-year LRC rates 67.5% and 54.7%, respectively). However, with a median PFS of 10.8 months, median DMFS of 11.6 months and median OS of only 18 months, the oncologic outcomes for this elderly patient population are considerably worse than for the highly selected younger cohorts of patients that have defined treatment standards based on several large randomized controlled trials in recent years (19, 20). For example, the recently published CheckMate 577 trial reported a disease-free survival of 22 months for patients with completely resected stage II or III cancers of the esophagus or gastroesophageal junction when patients received additional adjuvant treatment with nivolumab for pathologically incomplete remission after neoadjuvant chemoradiation (21). In contrast, several retrospective studies with large patient cohorts suggested that the OS among older patients might be comparable to that of younger patients after multimodal treatment including surgery (14, 15). As the majority of patients in our analysis was classified as technically or conditionally unresectable, the oncologic outcomes in our study should be compared more with results from other trials in which definitive chemoradiation was applied. In this regard, several landmark trials of definitive chemoradiation in patients with ESCC reported comparable median OS rates ranging between 14 to 19 months (22–24).

In our analysis, unmodified administration of chemotherapy in combination with radiation and neoadjuvant chemoradiotherapy followed by surgery were found to be significant prognosticators for PFS and OS. In addition, administration of standard chemoradiotherapy without dose reduction resulted in improved DMFS, whereas none of the analyzed clinico-pathological factors had a statistically significant impact on LRC. Similarly to our previous trials, we could not demonstrate age- or comorbidity-related differences in terms of outcome (25–28).

Several other recently published analyses with exclusively elderly patients also demonstrated the prognostic value of concomitant chemoradiation without dose reduction of chemotherapy (8, 9, 13, 16, 17, 29). However, there are also a few retrospective studies using propensity score matching that have reported no benefit of concomitant chemoradiation in elderly patients with esophageal cancer (30) or have demonstrated a benefit of chemoradiation only for patients with cT4 tumors, absence of nodal involvement (cN0), or diabetes (18). Given these conflicting results, further large multi-center analyses are needed to clarify the role of concomitant chemoradiation in elderly esophageal cancer patients in the definitive or neoadjuvant therapy setting (31).

Beyond concomitant chemotherapy, other retrospective studies found additional prognostic factors for OS after radiation treatment of elderly ESCC patients such as T and N stages, early tumor stage, treatment response, or nutritional status (13, 17, 29, 32, 33).

For definitive treatment, radiotherapy was prematurely discontinued in 10% of patients, and the full treatment regimen of definitive chemoradiotherapy including all concomitant and adjuvant chemotherapy cycles could only be administered to 41% of patients due to treatment-related toxicities. Definitive radiotherapy alone was performed in 19% of the patients because they were classified as unfit for concurrent chemotherapy. Compared with the RTOG 8501, ARTDECO, or PRODIGE5/ACCORD17 trials, treatment compliance was substantially worse in our older patient population, although severe and life-threatening adverse events were documented less frequently (5, 20, 34, 35). As an explanation for this discrepancy, treatment de-escalation in older patients is more readily performed in case of mild-to-moderate acute side effects than in younger patients.

In the neoadjuvant setting, treatment adherence was substantially better in our analysis with 71% of patients receiving the full treatment regimen of concomitant chemoradiation. Nevertheless, complete treatment delivery was still considerably worse in our elderly study population compared to the CROSS trial (6). Additionally, in our dataset, dose reduction of chemotherapy in the setting of concomitant chemoradiotherapy was relatively common. In this context, we demonstrated that definitive (chemo)radiotherapy, dose escalation with brachytherapy, and stent implantation were important baseline factors significantly associated with severe treatment-related acute toxicities.

Albeit our analysis provides comprehensive data on treatment adherence, toxicity and outcome in one of the largest multi-center cohorts of elderly ESCC patients undergoing neoadjuvant or definitive chemoradiation, it has limitations due to its retrospective character. For example, detailed information on concomitant diseases and data on clinical factors such as patients’ nutritional status, laboratory parameters (e.g., CRP levels or renal function at baseline and during treatment), or smoking status could not be systematically collected. Furthermore, patients’ quality of life could not be assessed retrospectively and requires further prospective investigation. Retrospective evaluation of the general condition may have a high interobserver variability, thus geriatric assessments including many different domains of life of elderly patients such as functional, nutritional, cognitive, psychosocial and socioeconomic status may provide a more reliable assessment of patient performance (36). The ability of geriatric assessments to predict chemotherapy-associated toxicities has been shown in other cancers, so the relevance of geriatric assessments should be further addressed in future prospective studies (37, 38).

## Conclusion

In summary, our multi-center analysis of 161 elderly ESCC patients indicates that chemoradiotherapy results in respectable LRC but relatively low OS and, in a substantial proportion of elderly patients, treatment-related acute toxicities which required dose reduction of radiotherapy and/or chemotherapy in the setting of neoadjuvant or definitive (chemo)radiotherapy. We demonstrated that concomitant chemoradiation without dose reduction of chemotherapy is the key prognostic factor for improved PFS, DMFS and OS of elderly patients with SCC of the esophagus. Therefore, it is of strong importance to carefully select those patients suitable for standard treatment, and further analyses are required to identify predictive factors for the tolerability of concurrent systemic treatment in elderly patients.

## Data availability statement

The original contributions presented in the study are included in the article/**Supplementary Material**. Further inquiries can be directed to the corresponding author.

## Ethics statement

The studies involving human participants were reviewed and approved by Ethics committees of the medical faculties of the universities of Mainz (no reference number), Freiburg (reference no. 275/18) and Heidelberg (reference no. S-040/2018). Written informed consent for participation was not required for this study in accordance with the national legislation and the institutional requirements.

## Author contributions

TB, SA, DW, and NN developed and planned the analysis. DW is responsible for statistical considerations/basis of the analysis. TB, SA, AM, EN, DW, MMu, SK, AR, A-LG, JD, CF, MMö, PG, HS, and NN participated in data collection and/or interpretation of the results. TB and NN wrote the manuscript. All authors contributed to the article and approved the submitted version.
