# Supplementary material for: Chemoradiotherapy in geriatric patients with squamous cell carcinoma of the esophagus: Multi-center analysis on the value of standard treatment in the elderly
Source: Front Oncol. 2023 Mar 3;13:1063670. doi: 10.3389/fonc.2023.1063670 (PMC10022427; doi:10.3389/fonc.2023.1063670)
Supplement: Supplementary file 1 [file Table_1.docx]

**Supplemental file 1:**

**Table S1** Chemotherapy regimens concurrent with neoadjuvant radiotherapy

| **Chemotherapy regimens** | **n** | **%** |
| --- | --- | --- |
| Cisplatin (20 mg/m^2^ of body surface area) d1-5 and 5-FU (1000 mg/m^2^ of body surface area) d1-5 at weeks 1, 5, 9 and 13 | 12 | 28.6 |
| Cisplatin (75 mg/m^2^ of body surface area) d7 and 5-FU (15 mg per kilogram of body weight) d1-5 at weeks 1 and 6 | 11 | 26.2 |
| Paclitaxel (50 mg/m^2^ of body surface area) and Carboplatin (area under the curve of 2 mg/ml/min) d1, 8, 15, 22 and 29 | 19 | 45.2 |

**Abbreviations:** 5-FU = 5-fluorouracil, d = day, n = number of patients
