# Supplementary material for: Chemoradiotherapy in geriatric patients with squamous cell carcinoma of the esophagus: Multi-center analysis on the value of standard treatment in the elderly
Source: Front Oncol. 2023 Mar 3;13:1063670. doi: 10.3389/fonc.2023.1063670 (PMC10022427; doi:10.3389/fonc.2023.1063670)
Supplement: Supplementary file 2 [file Table_2.docx]

**Supplemental file 2:**

**Table S2** Chemotherapy regimens concurrent with definitive radiotherapy

| **Chemotherapy regimens** | **n** | **%** |
| --- | --- | --- |
| Cisplatin (75 mg/m^2^ of body surface area) d1 and 5-FU (1000 mg/m^2^ of body surface area) d1-4 at weeks 1, 5, 8 and 11 | 21 | 17.6 |
| Cisplatin (20 mg/m^2^ of body surface area) d1-5 and 5-FU (600 mg/m^2^ of body surface area) d1-5 at weeks 1 and 5 | 13 | 10.9 |
| Cisplatin (20 mg/m^2^ of body surface area) d1-5 and 5-FU (1000 mg/m^2^ of body surface area) d1-5 at weeks 1, 5, 9 and 13 | 22 | 18.5 |
| Cisplatin (75 mg/m^2^ of body surface area) d7 and 5-FU (15 mg per kilogram of body weight) d1-5 at weeks 1 and 6 | 3 | 2.5 |
| Paclitaxel (50 mg/m^2^ of body surface area) and Carboplatin (area under the curve of 2 mg/ml/min) d1, 8, 15, 22, 29 and 36 | 10 | 8.4 |
| Oxaliplatin (85mg/m^2^ of body surface area), leucovorin (200 mg/m^2^ of body surface area) and 5-FU (400 mg/m^2^ of body surface area) bolus on day 1 followed by 5-FU (1600 mg/m^2^ of body surface area) 46 hours continuous infusion (FOLFOX) at weeks 1, 3, 5, 7, 9 and 11 | 9 | 7.4 |
| Mitomycin C (10 mg/m^2^ of body surface area) d1 and 5-FU (1000 mg/m^2^ of body surface area) d1-4 at weeks 1, 5, 8 und 11 | 2 | 1.7 |
| Carboplatin and 5-FU* | 4 | 3.4 |
| Capecitabine or 5-FU alone* | 6 | 5.0 |
| Cisplatin or Carboplatin alone* | 2 | 1.7 |

* = no informations on dosage and administration.

**Abbreviations:** 5-FU = 5-fluorouracil, d = day, n = number of patients
