# Supplementary material for: Chemoradiotherapy in geriatric patients with squamous cell carcinoma of the esophagus: Multi-center analysis on the value of standard treatment in the elderly
Source: Front Oncol. 2023 Mar 3;13:1063670. doi: 10.3389/fonc.2023.1063670 (PMC10022427; doi:10.3389/fonc.2023.1063670)
Supplement: Supplementary file 3 [file Table_3.docx]

**Supplemental file 3:**

**Table S3** Recurrence patterns of elderly patients after definitive or neoadjuvant (chemo)radiotherapy.

| **Variable** | **Value** | **n** | **%** |
| --- | --- | --- | --- |
| Locoregional relapse | yes | 43 | 26.7 |
|  | no | 118 | 73.3 |
| Local relapse | yes | 40 | 24.8 |
|  | no | 121 | 75.2 |
| Progressive disease after neoadjuvant or definitive (chemo)radiotherapy | yes | 124 | 77.0 |
|  | no | 37 | 23.0 |
| Distant metastases | yes | 41 | 25.5 |
|  | no | 120 | 74.5 |
| Liver metastases | yes | 6 | 3.7 |
|  | no | 155 | 96.3 |
| Lung metastases | yes | 23 | 14.3 |
|  | no | 138 | 85.7 |
| Bone metastases | yes | 7 | 4.3 |
|  | no | 154 | 95.7 |
| Brain metastases | yes | 1 | 0.6 |
|  | no | 160 | 99.4 |
| Kidney metastases | yes | 2 | 1.2 |
|  | no | 159 | 98.8 |
| Peritoneal carcinomatosis | yes | 2 | 1.2 |
|  | no | 159 | 98.8 |
| Other distant metastases | yes | 9 | 2.5 |
|  | no | 152 | 97.5 |
