# Supplementary material for: Chemoradiotherapy in geriatric patients with squamous cell carcinoma of the esophagus: Multi-center analysis on the value of standard treatment in the elderly
Source: Front Oncol. 2023 Mar 3;13:1063670. doi: 10.3389/fonc.2023.1063670 (PMC10022427; doi:10.3389/fonc.2023.1063670)
Supplement: Supplementary file 4 [file Table_4.docx]

**Supplemental file 4:**

**Table S4** Analysis of clinicopathological factors to predict the occurrence of higher-grade acute toxicities

| **Variable** | **OR** | **95% CI** | **p-value** |
| --- | --- | --- | --- |
| Age | 1.01 | 0.95 - 1.09 | 0.70 |
| Female gender | 1.09 | 0.49 - 2.45 | 0.83 |
| ECOG | 1.00 | 0.97 - 1.03 | 1.00 |
| CCI | 1.23 | 0.99 - 1.53 | 0.07 |
| Localization upper thoracic third | 0.30 | 0.09 - 1.02 | 0.17 |
| Localization middle thoracic third | 0.32 | 0.10 - 1.03 | 0.17 |
| Localization lower thoracic third | 0.23 | 0.07 – 0.82 | 0.17 |
| Tumor length | 1.01 | 0.88 - 1.16 | 0.86 |
| cT2 | 2.40 | 0.21 - 27.72 | 0.43 |
| cT3 | 1.08 | 0.11 - 10.89 | 0.43 |
| cT4 | 2.18 | 0.19 - 25.02 | 0.43 |
| cN1 | 0.41 | 0.17 - 0.95 | 0.14 |
| cN2 | 0.48 | 0.16 - 1.43 | 0.14 |
| cN3 | 2.63 | 0.22 - 31.57 | 0.14 |
| Tumor stage UICC 2 | 1.33 | 0.11 - 16.14 | 0.28 |
| Tumor stage UICC 3 | 0.59 | 0.05 - 6.97 | 0.28 |
| Tumor stage UICC 4a | 1.10 | 0.09 - 13.55 | 0.28 |
| Stent implantation | 2.58 | 1.06 - 6.28 | **0.04** |
| Brachytherapy | 4.38 | 1.70 - 11.31 | **0.002** |
| Chemotherapy without dose reduction | 0.50 | 0.16 - 1.57 | 0.48 |
| Neoadjuvant CRT followed by surgery | 0.07 | 0.01 - 0.29 | **< 0.001** |
| Switch from neoadjuvant (C)RT to definitive (C)RT | 1.63 | 0.41 – 6.55 | **< 0.001** |

**Abbreviations:** ECOG = Eastern Cooperative Oncology Group, CCI = Charlson Comorbidity Index, UICC = Union for International Cancer Control, (C)RT = (chemo)radiotherapy. **Bold values = significant p-values.**
