# Supplementary material for: Chemoradiotherapy in geriatric patients with squamous cell carcinoma of the esophagus: Multi-center analysis on the value of standard treatment in the elderly
Source: Front Oncol. 2023 Mar 3;13:1063670. doi: 10.3389/fonc.2023.1063670 (PMC10022427; doi:10.3389/fonc.2023.1063670)
Supplement: Supplementary file 5 [file Table_5.docx]

**Supplemental file 5:**

**Table S5** Analysis of clinicopathological factors to predict the occurrence of higher-grade chronic toxicities

| **Variable** | **OR** | **95% CI** | **p-value** |
| --- | --- | --- | --- |
| Age | 1.07 | 0.98 - 1.17 | 0.11 |
| Female gender | 1.27 | 0.44 - 3.62 | 0.66 |
| ECOG | 0.95 | 0.92 - 0.99 | **0.01** |
| CCI | 1.06 | 0.79 - 1.42 | 0.69 |
| Localization upper thoracic third | 0.70 | 0.15 - 3.36 | 0.94 |
| Localization middle thoracic third | 0.69 | 0.16 - 3.05 | 0.94 |
| Localization lower thoracic third | 0.56 | 0.11 - 2.86 | 0.94 |
| Tumor length | 1.01 | 0.84 - 1.21 | 0.96 |
| cT2 | NA | NA | NA |
| cT3 | NA | NA | NA |
| cT4 | NA | NA | NA |
| cN1 | 2.50 | 0.66 - 9.39 | 0.39 |
| cN2 | 0.94 | 0.15 - 6.09 | 0.39 |
| cN3 | 5.67 | 0.39 - 82.24 | 0.39 |
| Tumor stage UICC 2 | NA | NA | NA |
| Tumor stage UICC 3 | NA | NA | NA |
| Tumor stage UICC 4a | NA | NA | NA |
| Stent implantation | 0.84 | 0.22 - 3.12 | 0.79 |
| Brachytherapy | 2.16 | 0.69 - 6.79 | 0.20 |
| Chemotherapy without dose reduction | 0.27 | 0.07 - 1.01 | 0.11 |
| Neoadjuvant CRT followed by surgery | NA | NA | NA |
| Switch from neoadjuvant (C)RT to definitive (C)RT | 4.27 | 1.00 - 18.23 | **< 0.001** |

**Abbreviations:** NA = not analyzable, ECOG = Eastern Cooperative Oncology Group, CCI = Charlson Comorbidity Index, UICC = Union for International Cancer Control, (C)RT = (chemo)radiotherapy. **Bold values = significant p-values.**
